# Supplementary material for: Modeling Chikungunya control strategies and Mayaro potential outbreak in the city of Rio de Janeiro
Source: PLoS One. 2020 Jan 28;15(1):e0222900. doi: 10.1371/journal.pone.0222900 (PMC6986714; doi:10.1371/journal.pone.0222900)
Supplement: Attachment — Submitted filename: Response_to_Reviewers-PLOSONE-MOSQ.pdf [file pone.0222900.s002.pdf]

Response Letter for the manuscript entitled: “Modeling Chikungunya Control Strategies and Mayaro Potential Outbreak in the city of Rio de Janeiro”

We want to thank the reviewers for reading the manuscript and providing thoughtful evaluations, including several suggestions on how to improve this work. In the revised version of the manuscript and the present response letter, we have carefully addressed all suggestions. Corrections and additions in the revised manuscript are marked in red color. Please find below our responses and a description of the revision after the comments of the reviewers:

Reviewer #1: “*Authors are presenting a mathematical epidemiology modeling approach to describe outbreaks of Chikungunya in Rio de Janerio, Brazil for the years 2016, 2018, and 2019. Parameters for the Chikungunya model are obtained from the published literature and unknown parameters are estimated using the best fit to the solved equation. Then, parameters for Mayaro virus are plugged in the equation in order to calculate the basic reproduction number for Mayaro in the same geographic region. Finally, for Chikungunya in the year 2018, the authors developed equations resembling three vector control strategies.*
The article is well written and their modeling is a book approach to vector-borne disease mathematical modeling. Although well developed, the paper lacks a broader discussion of their findings or lack of them. First, the title of the manuscript is not reflecting what is done in the manuscript, specifically; intervention estimations are done only for Chikungunya in 2018, so Mayaro is never considered apart of the R0 estimation.”
Authors’ comments: The authors agree with the reviewer’s comments. The title was modified to: ”Modeling Chikungunya Control Strategies and Mayaro Potential Outbreak in the city of Rio de Janeiro”

Reviewer #1: “*Considering the Mayaro model, authors are replacing Chikungunya parameters by those described for Mayaro and then presented results for this pathogen. Although this is valid, the manuscript lacks any other discussion on the topic, this is key in order to consider this manuscript as a scientific contribution worth to be published in a journal, otherwise, we can also fit the parameters for any vector-borne disease (let’s say, Eastern Equine encephalitis, West Nile, etc) and comment on the basic reproduction number. Mayaro specifically grants discussion regarding its life sylvatic/urban life cycle, its endemicity in Latin America (it has been present in the continent longer than Chikungunya), its reservoirs, among others. Further, from the infectious diseases dynamics perspective, is more similar to Yellow fever, thus, the taxonomic justification (Chikungunya and Mayaro are alphaviruses) is short to explain its dynamics in any particular region.*”
Authors’ comments: The reviewer is correct when he points out the similarity between Mayaro and Yellow Fever from the disease's dynamics perspective when the mosquito in consideration belongs to the *Haemagogus* genus (*Receveur, M. C., et al. (2010)*). In this work, it was considered the recent studies regarding the capability of urban vectors such as Aedes aegypti

(*Long KC et al. (2011), Kantor AM, et al. 2019,*) to transmit Mayaro. Among Chikungunya, Zika, and Dengue (which are epidemic in Rio de Janeiro), the choice of modeling Mayaro based on Chikungunya for taxonomic proximity is reasonable. The mathematical modeling presents the potential of Mayaro to be an epidemic in the city of Rio de Janeiro. The parameters related could be different in other cities and regions, which can be the difference whether the disease will be epidemic or not, mathematically speaking.

Reviewer #1: "*The model is also lacking uncertainty measures. Parameters for the Chikungunya model include ranges for the human and mosquito latent periods. Are the authors using the median of this range? Which are the uncertainties associated with their simulated best-fit distribution of cases presented in Fig 1? Which are the most sensitive parameters in their model? (Sensitivity analysis?)*"
Authors' comments: It was included three different sensitivity analyses (one in the main text and two in the Supporting Information). The first analysis presents a sampling of the parameter space ±5% around the best fit values by using a 100 thousand Markov-Monte Carlo simulations. It was compared the total number of people infected and the peak shift with the available surveillance data estimating the sensitivity by using the Spearman correlation between the quantity of interest and the parameter values. The second analysis presented is a time-dependent sensitivity analysis which is then integrated globally and also through three stages: from day 0 to day 120, from day 120 to day 180 and from day 180 to day 365. For this analysis, the derivative of the number of cases of infected people corresponding to each parameter is the measurement of its sensitivity. The third sensitivity analysis performs a variation of ±10% from the best-fit value every parameter alone while leaving the others as the best-fit set of values. The values of uncertainties were included in the main text.

Reviewer #1: "*I believe, the paper could be very useful in a journal considering 'teaching corners' since its model implementation is not novel enough but is well explained and developed. It can also be deployed with its code for further teaching applicabilities.*"
Authors' comments: The codes and tables of the surveillance data are provided in the supplementary material.

Reviewer #2: "*It is a well structured paper, clear and easy to read. Authors recognize that models provide insights in the behaviour of epidemics and help further discussions of goverment policies, but do not show real data.*"
Authors' comments: The reviewer is correct. The public interventions are simulated scenarios once there is not available surveillance data incorporating these government policies.
